# Astragaloside IV Improves Diabetic Kidney Disease by Regulating NLRP3 Inflammasome

**DOI:** 10.1155/jdr/3340719

**Published:** 2025-11-30

**Authors:** Han Zhu, Liping Zheng, Keqin Zhao, Xin Wang, Zeming Ma, Ao Zeng, Weijie Zhao, Wantong Zhang, Lu Han, Yong Huang

**Affiliations:** ^1^Jiangxi University of Chinese Medicine, Nanchang, Jiangxi, China; ^2^Institute of Basic Theory for Chinese Medicine, China Academy of Chinese Medical Sciences, Beijing, China; ^3^Institute of Clinical Pharmacology, Xiyuan Hospital, China Academy of Chinese Medical Sciences, Beijing, China; ^4^NMPA Key Laboratory for Clinical Research and Evaluation of Traditional Chinese Medicine, Beijing, China; ^5^Beijing Engineering Research Center of Printed Electronics, Beijing Institute of Graphic Communication, Beijing, China; ^6^Department of Nephrology, Affiliated Hospital of Jiangxi University of Traditional Chinese Medicine, Nanchang, Jiangxi, China

**Keywords:** astragaloside IV, diabetic kidney disease, inflammation, metabolic diseases, molecular mechanisms, natural products, NLRP3 inflammasome

## Abstract

Diabetic kidney disease (DKD) is a chronic complication that seriously affects the prognosis of diabetic patients and is a primary reason for end-stage renal disease worldwide. The existing treatment strategies have shown unsatisfactory results in the clinical practice of DKD, and there is an immediate need to discover novel and efficacious medicines. Natural products are considered to have the potential for drug design and development due to their diverse pharmacological effects. Astragaloside IV (AS-IV) has a variety of biological activities as a natural product, and existing studies have demonstrated that it can effectively delay the progression of DKD through multiple pathways. Aseptic inflammation is a key characteristic of DKD and is crucial in its pathogenesis. Nucleotide-binding oligomerization domain-like receptor protein 3 (NLRP3) inflammasome is an essential regulator of inflammatory amplification. In this review, we focus on the mechanisms by which AS-IV improves DKD by regulating NLRP3 inflammasome activation, including anti-inflammatory, antioxidative stress, reducing endoplasmic reticulum stress (ERS), regulating lipid metabolism disorders, and reducing pyroptosis. These discoveries have provided new ideas for the treatment of DKD.

## 1. Introduction

About one-third of people with diabetes may develop chronic microvascular complications of the kidneys, known as diabetic kidney disease (DKD) [1, 2]. As one of the most critical complications affecting mortality in patients with diabetes, DKD is also a major driver of chronic kidney disease (CKD) or end-stage renal disease, posing an undeniable burden on the healthcare system [3, 4]. The pathogenesis of DKD involves multiple independently acting but overlapping mechanisms and is extremely complex. Existing studies involve genetic factors, hyperglycemia, dyslipidemia, hemodynamic abnormalities, mitochondrial dysfunction, etc. [5–9]. One of the crucial features of DKD is aseptic inflammation, and the accumulation of proinflammatory cytokines and chemokines in the kidneys is a significant cause of renal dysfunction [10]. So, the critical role of inflammation in the development of DKD is a consensus among researchers. As a complex cascade of amplified responses, activation of the inflammatory response involves multiple cells and molecules [11]. Available studies suggest that nucleotide-binding oligomerization domain-like receptor protein 3 (NLRP3) inflammasome is strongly related to the progression of DKD.

The wide range of biological activities and therapeutic potential has led to an increasing number of studies focusing on natural products [12, 13]. A large body of evidence suggests that natural products have diverse nephroprotective effects, including but not limited to antioxidant, antifibrotic, or antiapoptotic [14–16]. Used as a traditional Chinese medicine, Radix Astragali (RA) is derived from the dried root of *Astragalus membranaceus*, a leguminous plant, and contains a variety of pharmacological components such as polysaccharides, saponins, and flavonoids [17]. Studies have shown that RA can attenuate renal injury to slow the progression of DKD and improve renal outcomes through a multipathway, multitarget mechanism independent of glucose lowering [18, 19]. With the widespread use of RA injections, RA tablets, and granules, extensive clinical trials have also demonstrated that RA preparations can effectively reduce urinary protein and serum creatinine in patients with DKD [20]. Among the aforementioned pharmacological components, astragaloside has the highest proportion in saponins [21]. One of them, astragaloside IV (AS-IV), is the most representative tetracyclic triterpenoid saponin due to its multiple pharmacological activities. As a key active ingredient of RA and a quality control indicator of RA, AS-IV can improve DKD through diverse pharmacological effects, including resisting oxidative stress, restoring mitochondrial quality, reducing endoplasmic reticulum stress (ERS), alleviating fibrosis, etc. [22].

**Figure 1:**
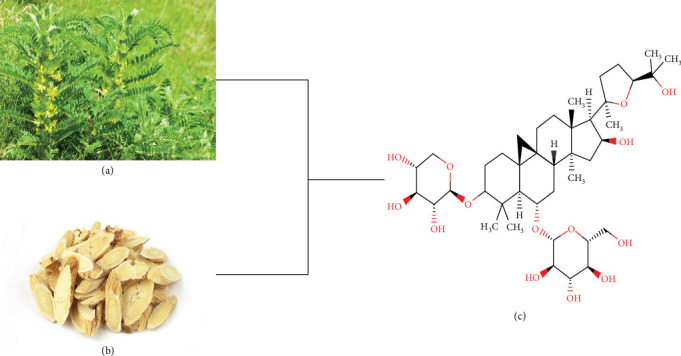
(a) *Astragalus membranaceus*. (b) RA. (c) Chemical structure of AS-IV.

The study of Shahzad et al. showed that the specific activation of NLRP3 inflammasome in podocytes is sufficient and necessary for DKD [23]. AS-IV can improve the DKD inflammatory response in db/db mice by suppressing the expression of NLRP3 inflammasome [24]. Current treatment strategies for DKD emphasize rigorous control of blood glucose, blood pressure, and lipids, and also include pharmacologic agents that specifically reduce albuminuria and slow kidney disease progression. Large randomized trials have demonstrated renoprotective effects for several classes—for example, sodium–glucose cotransporter-2 (SGLT2) inhibitors [25], nonsteroidal mineralocorticoid receptor antagonists [26], and, more recently, glucagon-like peptide-1 receptor agonists [27], all of which have shown beneficial effects on kidney outcomes. Some of these benefits are at least partly independent of glucose- or blood-pressure-lowering effects. Nevertheless, DKD continues to cause progressive renal dysfunction in many patients, and new therapies targeting inflammation, fibrosis, and other specific pathogenic pathways remain an important unmet need. Therefore, there is an urgent need for innovative and effective intervention drugs to slow down or reverse the progression of DKD. Here, we review the research reports of AS-IV improving DKD through NLRP3 inflammasome and summarize the results (Figure 1).

## 2. NLRP3 Inflammasome in DKD

**Figure 2:**
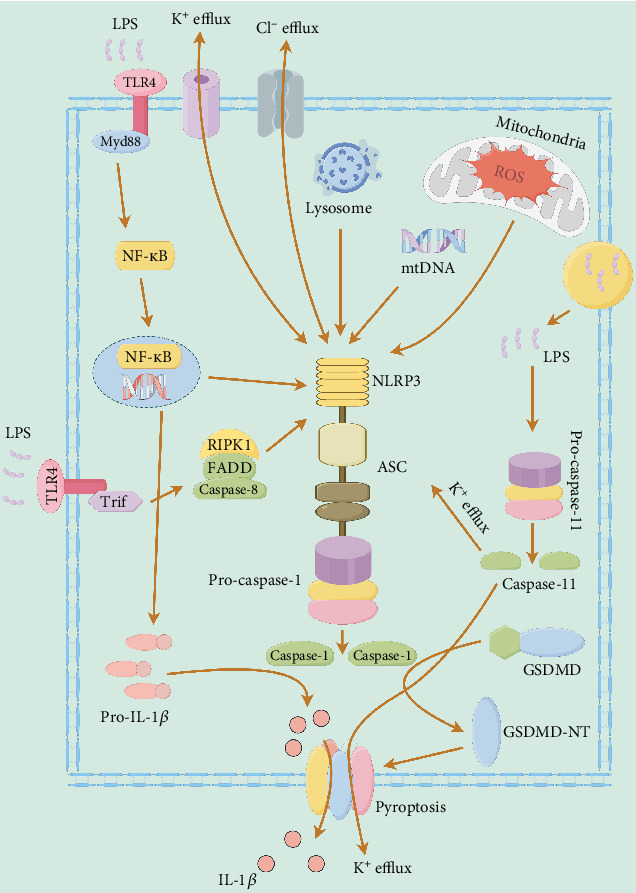
The mechanism of NLRP3 inflammasome activation.

NLRP3 inflammasome consists of the core protein of signal recognition and transduction: NLRP3, adaptor protein: apoptosis-associated speck-like protein containing a CARD (ASC), and inflammatory effector protein: caspase-1 [28]. NLRP3, as an innate immune pattern recognition receptor (PRR), recruits ASC after responding to stimulation and assembles caspase-1 to form the NLRP3 inflammasome. NLRP3 inflammasome in the activated state induces the maturation and release of interleukin-1*β* (IL-1*β*) and interleukin-18 (IL-18) to cause an inflammatory response [29]. In the meantime, caspase-1 is also activated and triggers gasdermin (GSDM)-mediated inflammatory cell death, namely, pyroptosis [30]. As a multiprotein complex that can recognize dangerous signals and respond to various stimuli, the normal existence of NLRP3 inflammasome is indispensable for the maintenance of intercellular immune homeostasis. The activation of NLRP3 inflammasome is divided into three ways (Figure 2, created using Figdraw): canonical, noncanonical, and alternative NLRP3 inflammasome activation. The canonical pathway needs to be initiated by inducing the expression of NLRP3 and pro-IL-1*β* through receptors such as TLR and NOD-like receptor (NLR). Then, the pathogen-associated molecular pattern (PAMP) or danger-associated molecular pattern (DAMP) triggers events such as oxidative stress to assemble and activate the inflammasome [31]. The noncanonical pathway is directly triggered by cytoplasmic lipopolysaccharide (LPS), which causes K^+^ efflux through the pannexin-1 channel, thereby activating the classical NLRP3 inflammasome [32]. The alternative pathway directly activates NLRP3 inflammasome only by a single TLR ligand signal without relying on other molecular or cellular events [33]. It should be noted that this alternative pathway cannot induce ASC to aggregate into ASC speck and lead to pyroptosis [34]. Although three activation pathways have been explored, the key upstream molecules of NLRP3 and the exact mechanism of stimulus perception remain to be further elucidated.

In addition to pyroptosis, existing studies have highlighted that NLRP3 inflammasome is also linked to multiple cell deaths, including apoptosis, necroptosis, and ferroptosis. Key pro-apoptotic factors, such as Bcl2-associated X protein (BAX), Bcl2-antagonist/killer protein (BAK), and caspase-3/7/9, can directly promote the assembly and activation of NLRP3 inflammasome by inducing mitochondrial damage, potassium efflux, or release of oxidized mitochondrial DNA (mtDNA), leading to IL-1*β* maturation and release [35]. Caspase-8, on the other hand, is a central paradoxical player linking apoptosis and inflammasome, and its specific effects are highly dependent on the cellular environment and stimulus signaling. Caspase-8 can promote NLRP3 activation and inflammation by cleaving gasdermin D (GSDMD), pannexin-1, or directly processing pro-IL-1*β*, and can also play an inhibitory role under specific conditions, such as in dendritic cells [36]. The core molecule of necroptosis, receptor-interacting protein kinase 3 (RIPK3), and its downstream effector, mixed lineage kinase domain-like protein (MLKL), are the core hubs connecting necroptosis and NLRP3 inflammation. Both of them can directly drive the assembly and activation of NLRP3 inflammasome by inducing oxidative stress, potassium outflow, and ASC speck formation through a variety of mechanisms, and this process can be independent of the pyroptosis executive protein GSDMD [37, 38].In addition, Z-DNA-binding protein 1 (ZBP1), which is involved in necroptosis, can act as an upstream sensor of RIPK3 to activate NLRP3 through the RIPK3-caspase-8 axis in specific pathogenic infection states, such as influenza viruses and certain fungi [39]. Glutathione peroxidase 4 (GPX4) and glutathione (GSH), the key antioxidant molecules of ferroptosis, apparently depressed the activation of NLRP3 inflammasome and the liberation of IL-1*β* by inhibiting oxidative stress and caspase-11-dependent pyroptosis [40, 41]. The substrate ubiquinone and its derivatives of ferroptosis suppressor protein 1 (FSP1), the upstream regulator of ferroptosis, also showed similar inhibitory effects [42]. Squalene, a characteristic metabolite of ferroptosis, has been found to be a potent activator of NLRP3 inflammasome, while the absence of ferroptosis inhibitor nuclear factor E2-related factor 2 (NRF2) blocks NLRP3 activation by inhibiting ASC speck formation and controlling phagocytosis [43, 44]. Although the mechanism between NLRP3 inflammasome and cell death has been partially elucidated, how to coordinate the relationship between multiple types of cell death and NLRP3 inflammasome activation needs to be further explored.

Progressive DKD is the common result of multiple types of regulated cell death (RCD). The genetic analysis of glomerular endogenous cells in DKD patients showed that there was obvious ferroptosis [45]. In the research of necroptosis and apoptosis of podocytes in the context of DKD, it was found that the former may make a bigger impact on podocyte injury than the latter [46]. Studies on the DKD mouse model have found that redox imbalance in renal cells can induce pyroptosis, which in turn aggravates renal cell damage and renal dysfunction [47]. Therefore, as the core target of pyroptosis, apoptosis, necroptosis, and ferroptosis, abnormally activated NLRP3 inflammasome contributes to the development of DKD. A variety of metabolites in the pathological process of DKD can activate NLRP3, such as excessive reactive oxygen species (ROS) induced by hyperglycemia [48]. With the activation of NLRP3 inflammasome, the downstream inflammatory effector molecules IL-1*β* and IL-18 will also increase, and biopsy shows that the expression level of both is positively correlated with the degree of renal dysfunction [49]. NLRP3 expression was significantly increased in the glomeruli of diabetic patients with proteinuria, and the same was observed in the kidneys of db/db mice [50]. Knockout of NLRP3 can significantly alleviate inflammation and fibrosis and improve renal function in the DKD mouse model [51]. In cellular experiments, siRNA treatment of the NLRP3 gene in human kidney (HK-2) cells in a high-glucose (HG) environment reduced apoptosis and ROS production [52]. Several in-depth mechanistic studies have demonstrated that NLRP3 is one of the central targets that exacerbate the progression of DKD, and that some molecules or proteins exert their biological effects by acting on NLRP3, examples include the ROS/thioredoxin (TRX)-interacting protein (TXNIP)/NLRP3 signal pathway [53], Toll-like receptor 4 (TLR4)/nuclear factor *κ*b (NF-*κ*B)/NLRP3 signal pathway [54], adenosine triphosphate (ATP)/P2X4/NLRP3 signal pathway [55], and C reactive protein (CRP)/Smad3/NLRP3 signal pathway [56]. It has been demonstrated that the activation of NLRP3 inflammasome is closely related to the onset and progression of DKD. It is a key factor in the pathological process of DKD and is essential for disease progression. Targeted regulation of NLRP3 inflammasome activation can effectively interfere with the development of DKD.

## 3. AS-IV Improves DKD by Acting on NLRP3 Inflammasome

**Figure 3:**
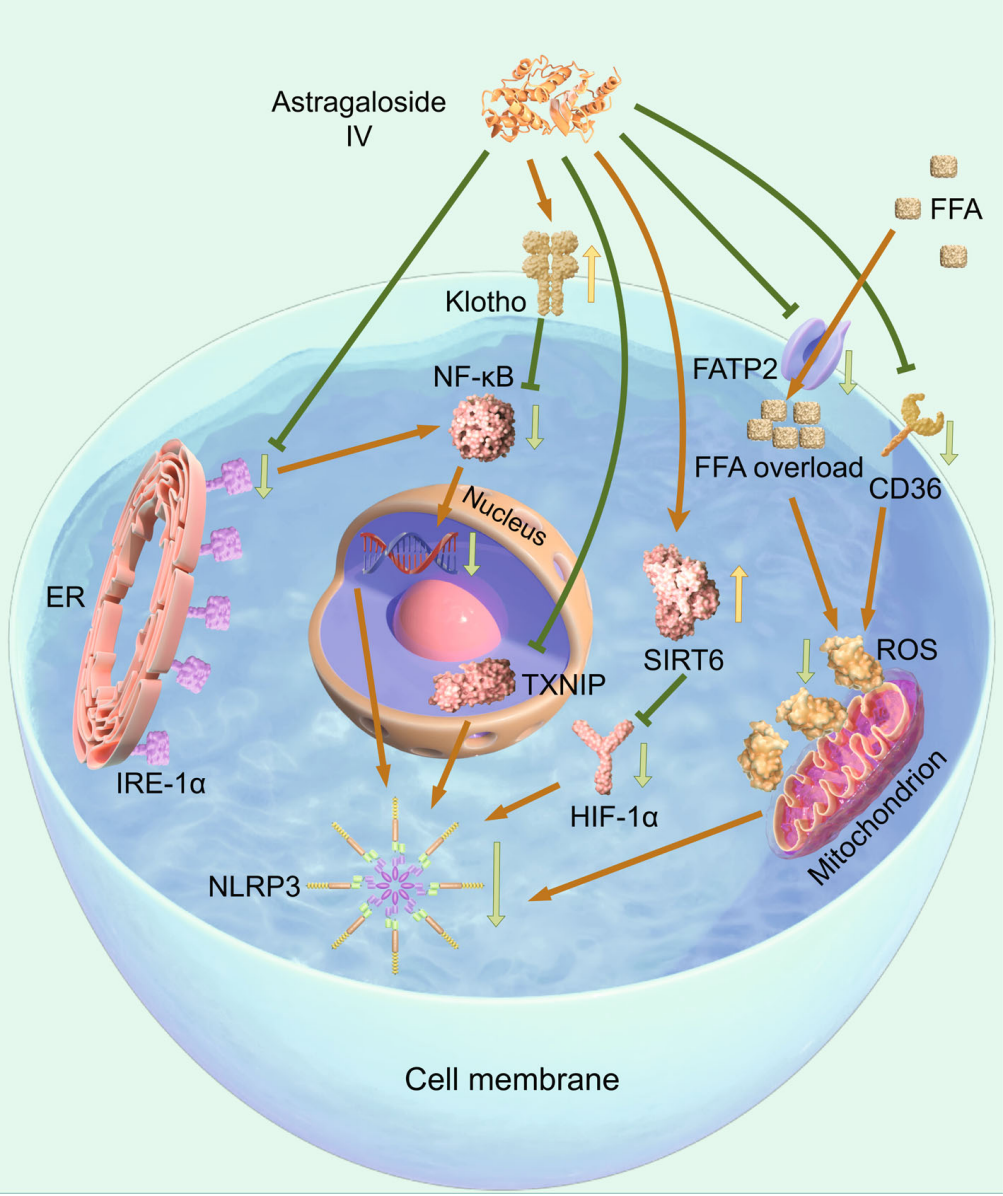
The mechanism of AS-IV improving DKD through NLRP3 inflammasome.

As a traditional medicinal plant, RA has significant renal protective activity, and its core bioactive substance AS-IV is the main component that mediates pharmacological effects. AS-IV regulates inflammatory response, metabolic homeostasis, and cell death through multiple targets, among which the inhibition of NLRP3 inflammasome is particularly critical. This component synergistically regulates the activation of NLRP3 inflammasome through multiple signal axes such as inositol-requiring enzyme 1*α* (IRE-1*α*), Klotho, fatty acid transporter 2 (FATP2), and TXNIP. It ultimately improves the inflammatory microenvironment and fibrosis pathology of DKD (Table 1 and Figure 3, created using FigDraw).

### 3.1. IRE-1*α*/NF-*κ*B/NLRP3 Signal Pathway

As an intracellular organelle, the endoplasmic reticulum (ER) can control the quality of protein production by promoting the correct folding of proteins [63]. A variety of adverse external stimuli can cause the correct protein structure to fail to form, which ultimately leads to the continuous accumulation of unfolded and misfolded proteins and triggers ERS [64]. IRE-1*α*, as a transmembrane protein that can detect ER activity, mediates the ERS process [65]. ERS induces unfolded protein response (UPR), followed by activation of IRE-1*α* to reduce abnormal protein accumulation and misfolding, thereby restoring cell homeostasis. Abnormally activated ERS phosphorylates IRE-1*α* and induces the production and release of large amounts of inflammatory factors [66]. NF-*κ*B, as a classic inflammation-related transcription factor, is composed of different subunits in the Rel protein family. In the context of cell homeostasis, NF-*κ*B protein is blocked by I*κ*B protein as an inhibitor and remains inactivated in the cytoplasm [67]. The phosphorylation and degradation of I*κ*B induced by external signal stimulation will release NF-*κ*B into the nucleus to initiate the transcription program. The transfer of NF-*κ*B to the nucleus will transcribe and activate NLRP3 [68]. In vitro experiments showed that AS-IV effectively alleviated HG-induced podocyte morphological damage and ER swelling, and significantly downregulated the expression of IRE-1*α*/glucose-regulated protein 78 (GRP78) ERS pathway and its downstream NLRP3 inflammasome and pyroptosis key factors. The effect was comparable to that of the IRE-1*α* inhibitor. In the DKD model, AS-IV significantly improved hyperglycemia, dyslipidemia, and renal dysfunction, and alleviated pathological changes such as glomerular hypertrophy, mesangial expansion, and cell proliferation. The protective mechanism is also dependent on the inhibition of IRE-1*α*/GRP78-mediated ERS and downstream NLRP3 inflammation and pyroptosis pathways. The DKD rat model established by streptozotocin (STZ) injection and high-fat diet (HFD) and in vitro podocyte experiments proved that AS-IV can alleviate renal pathological changes and renal function decline by regulating IRE-1*α*/NF-*κ*B/NLRP3 pathway, reduce ERS and inflammation, and improve podocyte pyroptosis to play a protective role [57].

### 3.2. Klotho/NF-*κ*b/NLRP3 Signal Pathway

Klotho proteins, initially of interest for their antiaging properties, serve as a coreceptor for fibroblast growth factor 23 (FGF23) and are expressed at much higher levels in the kidney than in other organs [69]. Based on existing research, Klotho protein deficiency plays a cardinal position in the onset and evolution of CKD [70]. Its deficiency directly drives renal tubular cell senescence, endothelial dysfunction, and impaired angiogenesis and mediates hyperphosphatemia and mineral metabolism disorders by interacting with the FGF23 pathway disorders [71]. In addition, low Klotho status constitutes a key pathological marker of CKD-related multisystem complications, such as accelerated cardiovascular disease [72], cognitive impairment [73], and salt-sensitive hypertension [74]. Existing studies have found that Klotho has anti-inflammatory, improves oxidative stress, and has other renal protective effects [75]. Klotho can inhibit NF-*κ*B and NLRP3 inflammasome, and the expression of Klotho itself is also regulated by NF-*κ*B [76]. At the in vivo level, AS-IV can improve the renal function of DKD rats, upregulate the levels of Klotho protein in the kidney and serum, and effectively inhibit the pyroptosis of glomerular cells. In vitro, AS-IV inhibited HG-induced podocyte pyroptosis and oxidative stress by upregulating Klotho expression, which was dependent on blocking the NF-*κ*B/NLRP3 inflammatory axis. Klotho gene knockdown could reverse the above protective effects, while antioxidants and NF-*κ*B/NLRP3 pathway inhibitors could simulate the pyroptosis inhibition effect of AS-IV. The outcome indicated that AS-IV attenuated podocyte damage in the HG background through the Klotho/NF-*κ*b/NLRP3 axis [58].

### 3.3. FATP2/ROS/NLRP3 Signal Pathway

FATP2 is a 70-kDa multifunctional protein that activates long-chain fatty acids (LCFAs) and transports LCFAs [77]. Studies have found that FATP2 is involved in the evolution of DKD. FATP2 plays a central pathogenic position in the lipotoxic injury of DKD. The abnormal reabsorption of albumin-bound long-chain fatty acids (FAs) at the top of renal tubules mediated by FATP2 is the key mechanism of lipid accumulation and renal injury in DKD [78]. The key evidence shows that the absence of FATP2 can significantly reverse the renal pathological changes, functional abnormalities, and hyperglycemia in DKD model mice, confirming that FATP2 is a key molecular target for the occurrence of DKD [79]. For example, renal tubular atrophy, glomerulosclerosis, and interstitial fibrosis were significantly improved in FATP2-deficient DKD mouse models [80]. ROS are a group of highly efficient oxidants containing oxygen, including hydrogen peroxide (H_2_O_2_), hydroxyl radicals (OH^−^), etc. [81]. The production of ROS in the HG environment can induce and aggravate oxidative stress in the kidney and accelerate the pathological changes and renal dysfunction of DKD [82]. AS-IV can dose-dependently reduce ROS levels and NLRP3-mediated inflammatory responses in DKD rat models and palmitic acid (PA)-treated NRK-52E cells. Treatment with FATP2 siRNA or FATP2 inhibitor can further enhance the role of AS-IV in protecting mitochondria and inhibiting inflammation. It can be seen that AS-IV can reduce renal tubular injury and improve the development of DKD by regulating the transport of FAs, namely, FATP2/ROS/NLRP3 axis [59].

### 3.4. CD36/ROS/NLRP3 Signal Pathway

CD36 is a member of the B2 scavenger receptor family and has multiligand recognition ability. Its binding targets include lipid molecules such as oxidized lipoproteins and long-chain FAs [83], as well as various protein ligands such as advanced glycation end products [84] and amyloid proteins [85]. The cell membrane expression of CD36 is dynamically regulated by insulin, HG, high fat, and other stimuli, involving the dual mechanism of endosomal translocation and new protein synthesis. Its expression level and subcellular localization directly affect cell metabolic homeostasis [86, 87]. As a multifunctional multiligand transmembrane glycoprotein receptor, CD36 is deeply involved in glucose and lipid metabolism, fibrosis, apoptosis, and inflammatory signal transduction [88]. CD36 expressed in a variety of renal cells can accelerate the development of DKD by promoting lipid deposition to produce oxidative stress and inducing an inflammatory response [89]. The application of AS-IV to the DKD rat model can be usefully applied to improve renal function and lesions, including tubulointerstitial inflammation and apoptosis of renal tubular epithelial cells, and inhibit the expression of CD36 and NLRP3. AS-IV treatment of PA-induced HK-2 cells reduced CD36-mediated lipid deposition and oxidative stress and inhibited NLRP3 activation while reducing CD36. It can be seen that AS-IV can consistently inhibit the CD36/ROS/NLRP3 inflammatory axis across the model, reduce kidney damage, and block the lipid accumulation-ROS-NLRP3 activation cascade [60].

### 3.5. TXNIP/NLRP3/GSDMD Signal Pathway

TXNIP is a regulator that directly activates TRX to maintain cellular redox balance [90]. As a core regulator of redox, TXNIP induces cell death by antagonizing TRX to increase ROS levels or promotes cell survival by stabilizing p53 to exert antioxidant effects. The contradictory functions of TXNIP constitute the molecular basis of complex phenotypes in diseases [91]. Current research shows that the role of TXNIP is not limited to regulating oxidative stress, but has various functions, such as activating ERS-mediated NLRP3 to further participate in the inflammatory process [92]. As a key node in the activation of NLRP3, TXNIP promotes the secretion of pro-inflammatory factors by activating the NF-*κ*B pathway through nuclear translocation in endothelial cells, while inhibiting TNF*α*-NF-*κ*B signaling in tumor cells to exert anti-inflammatory effects [93, 94]. It activates NLRP3 inflammasome through multiple pathways such as TRX1/TXNIP/ROS axis [95], stimulator of IFN genes (STING) interaction [96], C-X-C chemokine receptor type 4 (CXCR4) direct binding, and UPR-mediated mitochondrial dysfunction, and some mechanisms are independent of classical TRX1/TXNIP interaction and ROS regulation, highlighting the complexity of its inflammatory regulatory network [97, 98]. Blood, urine examination, and renal biopsy demonstrated a marked rise in the expression of TXNIP in DKD patients, and experiments showed that the absence of TXNIP could alleviate kidney damage in DKD mouse models, which proved that TXNIP played an instrumental position in DKD [99]. In vitro podocyte experiments confirmed that AS-IV inhibited HG-induced podocyte pyroptosis by blocking TXNIP-NLRP3 interaction. This effect can be mimicked and repeated by GSDMD silencing or NLRP3 inhibitor, and TXNIP-mediated pyroptosis activation can be reversed by AS-IV. Studies have found that AS-IV can play a renal protective role through the TXNIP/NLRP3/GSDMD signal pathway to delay the development of DKD [61].

### 3.6. Sirtuin 6 (SIRT6)/HIF-1*α*/NLRP3 Signal Pathway

SIRT6 is a member of the evolutionarily stable family of NAD^+^ dependent deacetylases and is involved in diverse cellular events such as metabolism, inflammation, apoptosis, and oxidative stress [100]. As a key epigenetic regulator, SIRT6 maintains systemic energy homeostasis by deacetylating histones and metabolic transcription factors, such as hypoxia inducible factor-1*α* (HIF-1*α*), forkhead box proteins O1 (FOXO1), peroxisome proliferator-activated receptor-*γ* coactivator-1*α* (PGC-1*α*), sterol regulatory-element binding proteins (SREBPs), and extensively integrating glycolipid metabolic pathways including inhibition of glycolysis and gluconeogenesis, enhancement of insulin sensitivity, promotion of FA oxidation and inhibition of lipid synthesis [101–103].In terms of inflammation, SIRT6 performs an anti-inflammatory function by targeting the NF-*κ*B signaling pathway by mediating histone H3 lysine 9 (H3K9) deacetylation, inhibiting RelA activity, and enhancing I*κ*B*α* expression [104]. At the same time, it regulates the intensity of inflammatory response by promoting the transcription, translation, and secretion of tumor necrosis factor-alpha (TNF-*α*) [105]. SIRT6 plays a crucial role in renal homeostasis. For example, SIRT6 deficiency accelerates renal injury in mice, especially podocyte foot process disappearance and progression of renal dysfunction [106]. Activation of HIF-1 is a common method for cells to adapt to a hypoxic environment [107]. HIF-1*α* is a subunit regulated by O_2_ in HIF-1. Existing studies have proven that it can deeply intervene in the progression of DKD by promoting capillary proliferation, inducing erythropoietin (EPO) expression, regulating oxidative stress, and promoting an inflammatory response [108]. After 8 weeks of AS-IV treatment on the STZ-induced DKD rat model, the expression of SIRT6 was significantly upregulated, and the expression of HIF-1*α* and NLRP3 was decreased. In vitro podocyte experiments also showed the same results after treatment with AS-IV in the context of HG, demonstrating that AS-IV can alleviate DKD progression by regulating the SIRT6/HIF-1*α*/NLRP3 axis to inhibit podocyte pyroptosis [62].

## 4. Conclusion and Perspectives

Taken together, the available studies suggest that AS-IV suppresses the initiation of NLRP3 inflammasome by modulating multiple signaling pathways, thereby delaying the development of DKD. The core mechanisms include inhibiting inflammatory response, antagonizing oxidative stress, reducing ERS, regulating lipid metabolism disorders, reducing pyroptosis, and improving glomerular podocyte and renal tubular epithelial cell injury. These studies of AS-IV provide a scientific basis for its development as a new therapeutic drug for DKD.

However, the pathogenesis of DKD presents a complex multitarget and multipathway regulatory network, and the mechanism of AS-IV intervention in DKD is still relatively rare. There is also a lack of comprehensive and in-depth research on specific mechanisms and molecular targets. Future research on AS-IV should focus on the role of multiple mechanisms such as autophagy, ferroptosis, post-translational modification, and mitochondrial function. In addition, most of the studies of AS-IV are obtained in the laboratory, and more clinical studies are needed to strengthen the clinical transformation of these findings, aiming to provide better treatment for DKD patients.

## Figures and Tables

**Table 1:** The mechanism of AS-IV improving DKD through NLRP3 inflammasome.

**Model**	**In vivo/in vitro**	**Mechanism**	**Pathways and targets**	**Reference**
HFD/STZ rats	In vivo	Anti-inflammatory, reduce ERS, reduce pyroptosis	Inhibition of IRE-1*α*/NF-*κ*B/NLRP3	[57]
HG-induced podocytes	In vitro			
HFD/STZ rats	In vivo	Anti-inflammatory, antioxidative stress, reduce pyroptosis	Upregulates Klotho and inhibits NF-*κ*B/NLRP3	[58]
HG-induced podocytes	In vitro			
HFD/STZ rats	In vivo	Anti-inflammatory, antioxidative stress, reduce renal tubular injury	Inhibition of FATP2/ROS/NLRP3	[59]
PA-induced NRK-52E cells	In vitro			
HFD/STZ rats	In vivo	Anti-inflammatory, antioxidative stress, regulate lipid metabolism, reduce pyroptosis	Inhibition of CD36/ROS/NLRP3	[60]
PA-induced HK-2 cells	In vitro			
db/db mice	In vivo	Anti-inflammatory, reduce pyroptosis	Inhibition of TXNIP/NLRP3/GSDMD	[61]
HG-induced podocytes	In vitro			
HFD/STZ rats	In vivo	Anti-inflammatory, antioxidative stress, reduce pyroptosis	Upregulates SIRT6 and inhibits HIF-1*α*/NLRP3	[62]
HG-induced podocytes	In vitro			

## Data Availability

The data used to support the findings of this study are included within the article.

## Nomenclature

DKD: diabetic kidney disease
AS-IV: astragaloside IV
NLRP3: nucleotide-binding oligomerization domain-like receptor protein 3
ERS: endoplasmic reticulum stress
RA: Radix Astragali
ASC: apoptosis-associated speck-like protein containing a CARD
PRR: pattern recognition receptor
IL-1*β*: interleukin-1*β*
IL-18: interleukin-18
GSDM: gasdermin
NLR: NOD-like receptor
PAMP: pathogen-associated molecular pattern
DAMP: danger-associated molecular pattern
LPS: lipopolysaccharide
BAX: Bcl2-associated X protein
BAK: Bcl2-antagonist/killer protein
mtDNA: mitochondrial DNA
GSDMD: gasdermin D
RIPK3: receptor-interacting protein kinase 3
MLKL: mixed lineage kinase domain-like protein
ZBP1: Z-DNA-binding protein 1
GPX4: glutathione peroxidase 4
GSH: glutathione
FSP1: ferroptosis suppressor protein 1
NRF2: nuclear factor E2-related factor 2
RCD: regulated cell death
ROS: reactive oxygen species
HK-2: human kidney
HG: high glucose
TXNIP: thioredoxin-interacting protein
TLR4: toll-like receptor 4
NF-*κ*B: nuclear factor *κ*b
ATP: adenosine triphosphate
CRP: C-reactive protein
IRE-1*α*: inositol-requiring enzyme 1*α*
FATP2: fatty acid transporter 2
ER: endoplasmic reticulum
UPR: unfolded protein response
STZ: streptozotocin
HFD: high-fat diet
FGF23: fibroblast growth factor 23
CKD: chronic kidney disease
LCFAs: long-chain fatty acids
FA: fatty acids
PA: palmitic acid
TRX: thioredoxin
STING: stimulator of IFN genes
CXCR4: C-X-C chemokine receptor type 4
SIRT6: sirtuin 6
HIF-1*α*: hypoxia inducible factor-1*α*
FOXO1: forkhead box proteins O1
H3K9: fistone H3 lysine 9
TNF-*α*: tumor necrosis factor-*α*
EPO: erythropoietin
